# Surgical Clinic of Prof. W. W. Dawson, at the Good Samaritan Hospital

**Published:** 1882-12-20

**Authors:** 


### SURGICAL CLINIC OF PROF. W. W. DAWSON AT THE GOOD SAMARITAN HOSPITAL.

### VARICOCELE—CASTRATION THE BEST REMEDY.

Mr. McN. is a farmer, has been annoyed for some time past by a condition in the scrotum known as varicocele. This is an enlarged, dilated and tortuous mass of veins, imparting the feeling of a "bag of worms" within the scrotum. Usually varicocele occurs upon the left side, the testicle hangs lower, is more dependent and the veins are larger than on the right side. There are anatomical as well as physical reasons for this difference. The left spermatic vein enters the left renal vein at a right angle, and has no valve to guard against downward pressure, hence its lifting power is greatly abridged. The right spermatic vein is supplied with valves in its course and does not enter the renal vein on that side, but empties direct into the vena cava; it is also guarded by a valve at its junction. Pressure of any kind upon the spermatic vessels when it interferes with venous return may produce varicocele. The disease often occurs in young men, being brought about by venereal excesses. In the case before you it follows a trauma—a blow upon the testicle.

The discomfort complained of from this tumor is a dull, heavy aching pain, running up to the groin along the cord and extends to the back; associated is a sensation of weight and dragging in the scrotum. These sufferings are mitigated by a recumbent posture, the patient is also relieved to some extent by the use of a suspensory bandage.

There is usually not much trouble in differentiating between varicocele and other scrotal tumors. Hydrocele has a regular outline, is elastic to the touch, and is translucent by transmitted light. Sarcocele is firm and globular. Scirrhus is hard and knotty and associated with a cachexia. Hernia travels from the ring downwards, pushing the testicle before it, it imparts to the sense of touch a doughy feel. Varicocele is an elongated tumor, irregular in shape, feeling like a "bag of worms," this symptom is nearly if not quite pathognomonic.

### TREATMENT.

These cases are seldom seen in the early stage, when attention to general health, bathing, etc., and the application of a suspensory bandage would not only relieve suffering but prevent the advance, the enlargement, the elongation of the veins. Young men are, however, very sensitive upon such matters. They seldom consult a physician until the veins are very large and the testicle atrophied.

This patient comes before us with a scrotum literally full of enlarged and tortuous veins with an atrophied, a blighted testicle. What is the best treatment? No disease of equal grade has had more devices suggested and adopted for relief than varicocele. No plan has yet been generally adopted, failures have been frequent. The operations may be grouped under three heads:

1. Obliteration of veins.
2. Ablation of scrotum.
3. Castration.

For the obliteration of the veins these vessels have been ligated with a great variety of ligatures, such as silk, metallic and animal. These ligatures have been applied subcutaneously, and again by incising the scrotum, exposing the veins before the application. The actual cautery has been drafted for the destruction of these tortuous venous canals, again electricity has been tried for the same purpose. One surgeon applies the ligatures firmly so as to obliterate at once the vessels, another proposes to do the same work gradually. Two objections have been urged and they are radical. First, in many cases phlebitis has followed and life lost; second, in cases where no inflammation has followed, ligation under all forms has failed to obliterate the veins—to obliterate them so as to relieve the suffering testicle from blood pressure. It may be said then that where obliteration of veins is not dangerous, no damage following the practice, it is unsatisfactory, it is inefficient.

ABLATION OF SCROTUM.

The object of this is to so abridge the scrotum as to pocket the testicle and shorten the veins. I have succeeded with this occasionally, but I sometimes have had disaster, and now only adopt it by the patient's selection. What real objections can be urged? The skin, in spite of all care, will, in some cases, slough and leave the testicle exposed. This is a serious matter, may result in loss of the organ, or, on account of the large surface exposed, pyæmia or tetanus may follow. When either of these giants appear I need not suggest to you the danger.

CASTRATION.

After a considerable experience I am decidedly of opinion that removal of the testicle, although apparently a radical operation, offers the best results. Ligation, as I have said, fails to obliterate the vessels. No surgeon would have temerity enough to include all the veins, those left soon enlarge and the scrotum is full of blood, and all the annoying emasculatory symptoms return.

There is one set of symptoms which have not been mentioned. I refer to a train of phenomena of the most distressing character, they are both moral and physical, direct and reflex; these especially pertain to those who attribute the affection to their own habits. A gloom settless over the poor invalid, he attributes every unpleasant sensation, every pain directly to this abnormality; this mental condition is reflected upon his system generally, he loses his appetite, is restless and sleepless; disturbed with exhausting dreams, he arises in the morning unrefreshed and wretched. An occasional nocturnal emission fills him with apprehension and fears of impotence. Under castration these distressing symptoms all disappear, plumpness takes the place of attenuated muscles, the skin loses its cold clamminess and becomes dry and warm, the palpitating heart becomes steady, the countenance assumes cheerfulness, the whole aspect of the man has changed, invalidism gives place to health.

In discussing this operation the question of sexual ability need not be entertained, the loss of a shrunken testicle like this is no loss—it is in fact a gain—the remaining organ with the balance of the system will be strengthened, its abilty for procreation reinforced.

This patient expected a less radical operation, hence shrinks from castration to-day, upon reflection he will doubtless adopt it.

The surgeon may conscientiously assure the sufferer that while castration offers relief most certainly, it is not more hazardous to life than less radical measures.—*Cincinnati Lancet and Clinic.*

